# Experimental and Theoretical Study of the Effects of Rare Earth Elements on Growth and Chlorophyll of Alfalfa (*Medicago sativa* L.) Seedling

**DOI:** 10.3389/fpls.2021.731838

**Published:** 2021-10-08

**Authors:** Kexiao Song, Jinzhu Gao, Shuo Li, Yunfu Sun, Haoyang Sun, Baiyu An, Tianming Hu, Xueqing He

**Affiliations:** College of Grassland Agriculture, Northwest A&F University, Yangling, China

**Keywords:** alfalfa, chlorophyll a, rare earth elements, quantum chemistry, lanthanides-chlorophyll

## Abstract

Rare earth elements (REEs) of low concentration are usually beneficial to plant growth, while they are toxic at high concentrations. The effects of treatment with lanthanum (La) (10 and 20 μM), cerium (Ce) (10 and 20 μM), and terbium (Tb) (10 and 20 μM) on seedling growth of alfalfa (*Medicago sativa* L.), which is one of the most important perennial leguminous forages in the world, were studied. The results showed that all three REE treatments quickened the germination of seeds. The length of shoot under La (20 μM) treatment was significantly shortened (*P* < 0.05). In addition, treatment with La, Ce, and Tb had a “hormesis effect” on root length. There was a significant decrease in chlorophyll content on treatment with the three REEs, and the degree of decline was in the order of La < Ce < Tb, under the same concentration. *In vitro* experiments and quantum chemical calculations were further performed to explain why the treatments with REEs reduced the chlorophyll content. *In vitro* experiments showed that La, Ce, and Tb treatments reduced the absorbance of chlorophyll, and the decrease followed in the order of La > Ce > Tb. Quantum chemical calculations predicted that the decrease in absorption intensity was caused by the reactions between La, Ce, Tb, and chlorophyll, which formed lanthanides-chlorophyll; and there were five types of stable lanthanides-chlorophyll. In conclusion, the decrease in chlorophyll content on treatment with REEs was caused by the change in chlorophyll structure.

## Introduction

Rare earth elements (REEs) are divided into two parts, light rare earth elements (LREE: cerium group elements) and heavy rare earth elements (HREE: yttrium group elements) (Binnemans et al., 2013), which are important plant growth stimulants in agriculture (Cotruvo, 2019; Kovaríková et al., 2019). Over the past decades, the application of REEs to plants has been investigated extensively (Chen, 2001; Hu et al., 2004; Lian et al., 2018; Yang et al., 2019; Fevzi et al., 2020). In 1980, when the field experiment started, China became the first country to use commercial rare earth fertilizers for crop production (Guo, 1985). Foliar spraying, seed treatment, fertilizer, and culture medium with REEs were carried out on a variety of crops, and the productivity of crops including wheat (*Triticum aestivum*) (Liang et al., 2005), soybean (*Glycine max*) (Cynthia et al., 2015), and other crops (Tommasi et al., 2020) increased by 5–15% (Xiong et al., 2000). It was found that REEs had a “hormesis effect” on plant growth and development (d'Aquino et al., 2009; Wen et al., 2011; Ochi et al., 2014). The hormesis effect is a dose-response relationship phenomenon characterized by low-dose stimulation and high-dose inhibition. So, a suitable concentration of REEs could stimulate plant growth and development (Hong et al., 2017), and improve photosynthetic capacity (García-Jiménez et al., 2017) and crop quality (Ramírez-Olvera et al., 2018). In the laboratory, the positive effects of REEs on crop production and plant physiological responses have been reported extensively (Liu et al., 2012). An appropriate amount of REEs promotes seed germination and root development (Ma et al., 2010). REEs could enter the chloroplast through clathrin-mediated endocytosis (CME), which required extracellular arabinogalactan proteins (AGPs) that were anchored on the outer face of the plasma membrane (Wang et al., 2019). In comparison to LREEs, HREEs with f electrons were more likely to bind to AGPs (Yang et al., 2018), and Turra (2018) found that LREEs were more fluid than HREEs. La (III) and Ce (III) are LREEs without f electrons, which were found to have a “hormesis effect” on plants (d'Aquino et al., 2009), while Tb (III) is a typical representative of HREEs with f electrons. Different REEs may have different effects on plants (Wang L. H. et al., 2014), and therefore we selected two representative types of REEs (LREE: La and Ce; HREE: Tb) for a comparative study. Alfalfa (*Medicago sativa* L.) is a perennial herb of leguminous forage and is also known as the “king of forage.” Alfalfa has the characteristics of long service life, high nutritional value, strong adaptability, and good economic and ecological benefits, so it is widely cultivated around the world (Krishna et al., 2020). Up to now, experiments with REEs on alfalfa have not been reported, and it is important to know how REEs affect the seedling growth of alfalfa.

Photosynthesis is an important aspect of the seedling growth of alfalfa. Photosynthesis is the physiological and material basis of plant growth (Xu et al., 2003), and it depends on chlorophyll. Chlorophyll contains a porphyrin ring, a central Mg ion, several sidechains, and a long hydrocarbon tail. There are two main types of chlorophyll, chlorophyll a and chlorophyll b. They differ only in the composition of the sidechain. The contents of chlorophyll a and chlorophyll b in wheat leaves increased with REEs (Chu, 1994). In rape (*Brassica napus* L.), the treatments with CeCl_3_ and Nd (NO_3_)_3_ increased the chlorophyll content by 9–40% (Jie and Yu, 1985). Our previous work showed that treatment with La increased the chlorophyll content of switchgrass (*Panicum virgatum* L.) (He et al., 2020). Shen et al. (1999) found that Nd^3+^ replaced Mg^2+^ in the center of the chlorophyll porphyrin ring, but there was no detailed information about coordination. Hong et al. (2001) found that the content of rare earth element-binding chlorophyll accounted for about 1–6% of the total chlorophyll in Osmunda foetida (*Dicranopteris linearis*), and speculated that La^3+^, Ce^3+^-chlorophyll was a double-layer structure, which was consistent with the experimental results *in vitro* (Hong et al., 2002). However, the most possible structure of lanthanides–chlorophyll and how the lanthanides–chlorophyll affects chlorophyll content of alfalfa are not clear yet.

Quantum chemical calculation is a powerful tool to explain the experimental phenomenon at the atomic level. It can predict the most stable structure and the most possible chemical reaction. It can also simulate the electronic absorption spectrum. With density functional theory (DFT), Liao et al. (2006) successfully predicted the molecular structures of lanthanide porphyrins. Otlyotov et al. (2020) optimized the molecular geometries of Ca and Zn complexes with porphyrazine. Yuriy et al. (2021) reported the molecular structures of Y, La, and Lu complexes with porphyrazine. Yin (2016) explored the possible reaction direction by calculating the Gibbs free energies of deprotonated and protonated species of porphyrin. With the time-dependent density functional theory (TD-DFT), Nemykin and Hadt (2010) simulated the electronic absorption spectra of ferrocenyl-containing porphyrins. Ziegler et al. (2013) calculated the electronic absorption spectra of N-confused porphyrins. Both the values were consistent with the experimental UV-vis spectra. In the present work, we employed quantum chemical calculations and *in vitro* experiments to study the possible substitution reactions between chlorophyll and REEs (La, Ce, and Tb).

Therefore, in this study, the effects of two different concentrations of REEs (La, Ce, and Tb) on alfalfa seedlings were investigated *in vivo*. To get insight into the reason behind why La, Ce, and Tb treatments affect chlorophyll content, *in vitro* experiments and quantum chemical calculations were adopted to explore the possible reactions between chlorophyll and REEs (La, Ce, and Tb). The purpose of this study was: (1) to reveal the effects of different REEs on alfalfa: seed germination, shoot length, root length, root activity, and chlorophyll content; (2) to predict the complex structures of REEs and chlorophyll, and explain the change in chlorophyll content based on the spectral property of the complexes.

## Materials and Methods

### Plant Material and Growth Conditions

The mature seeds of alfalfa were purchased from Xi'an BeeLoo Landscape Design Co., Ltd. (China) in July 2020. They were then cleaned and stored in paper bags at room temperature for later use. According to the International Seed Testing Association (2017), the thousand-seed weight was 2.257 g, and the seed viability was 97%. Chlorophyll a and chlorophyll b mixture (BR) was purchased from Shanghai source leaf Biotechnology Co. Ltd. (China). La(NO_3_)_3_·6H_2_O was purchased from Tianjin Guangfu Fine Chemical Research Institute (China), and its purity was more than 98%. Ce(NO_3_)_3_·6H_2_O was purchased from Afarsa (China) Chemical Co., Ltd. (China), and its purity was more than 99.99%. Tb (NO_3_)_3_·6H_2_O was purchased from Macklin company (China), and its purity was more than 99%.

### Germination Test

The seeds of alfalfa were randomly selected and then pre-cooled at 4°C for 24 h. After that, the seeds were disinfected with 75% alcohol for 30 s and washed five times with sterile water. There were seven treatments with La (NO_3_)_3_(10 μM, 20 μM), Ce(NO_3_)_3_(10 μM, 20 μM), Tb(NO_3_)_3_(10 μM, 20 μM), and H_2_O. Moreover, there were three samples in each treatment, and there were 50 seeds in each sample. For each treatment, the seeds were germinated in petri dishes on a two-layer filter paper imbibed with 5 ml of the corresponding solution, and then the seeds were put in a germination chamber at 25 ± 2°C, 85% RH, and a 16/8-h photoperiod (light/dark) with 10000Lx irradiance for 14 days. They were replenished with a specific quantity of water every day. Germination was defined as the emergence of the radical through the coat.


$$\begin{array}{l} \text{Germination rate }\left(\%\right)\\ \;=\frac{\text{Number of germinated seeds after n days}}{\text{Number of tested seeds}}\\ \;\times 100\%\left(\text{n}=4,\;14\right) \end{array}$$

### Seedling Growth Assay

After 14 days of seed germination, the shoot length, and root length of all the seedlings were measured. Root activity was determined by the triphenyl tetrazolium chloride (TTC) method (Ejazul et al., 2007). TTC is a redox pigment. Its solution is colorless, but it produces a red compound on reduction. The red compound is relatively stable, and therefore, TTC is widely used as a hydrogen acceptor for enzyme tests. For testing root activity, fresh root tissues (0.25 g) were first immersed into 10 ml phosphate buffer solution (1/15 mol/L) containing 0.4% (w/v) TTC and kept in the dark for 3 h at 37°C. Next, 2 ml H_2_SO_4_ (1 mol/L) was added. The roots were then dried and extracted with ethyl acetate. After that, the volume of red extract was increased to 5 ml by adding ethyl acetate. Finally, the absorbance of the red extract was measured at 485 nm. Root activity was calculated using the following equation:


$$\begin{array}{l} \text{Root activity }\left(\text{mg}.{\text{g}}^{-1}{\text{h}}^{-1}\right)=\frac{\text{amount of TTC reduction }\left(\text{μg}\right)}{\text{fresh root weight }\left(\text{g}\right)\times \text{time }\left(\text{h}\right)} \end{array}$$

To measure the chlorophyll content, a certain amount of fresh leaves was collected, and then they were ground for 60 s. After that, they were soaked in 2 ml 100% absolute ethanol for 24 h to extract chlorophyll. After centrifugation, the supernatant was adopted and its volume was increased to 7.5 ml by adding absolute ethanol. Finally, the absorbances of supernatant were determined at wavelengths of 665 nm and 649 nm. The values of chlorophyll content were expressed as mg/g of fresh weight (FW).


$$\begin{array}{l} \text{ Chl a}\left(\text{mg}/\text{L}\right)=13.70{\text{A}}_{665}-\;5.76{\text{A}}_{649}\\ \text{ Chl b}\left(\text{mg}/\text{L}\right)=25.80{\text{A}}_{649}-\;7.60{\text{A}}_{665}\\ \text{Chl total}\left(\text{mg}/\text{L}\right)=20.04{\text{A}}_{649}+\;6.10{\text{A}}_{665} \end{array}$$

Here, A_665_ and A_649_ are the absorbances of chlorophyll at 665 and 649 nm, respectively (Rowan, 1989; Ritchie, 2006).

### *In vitro* Experiments

The chlorophyll mixture [Mg-chlorophyll a (MgCA) and Mg-chlorophyll b (MgCB)] was dissolved in absolute ethanol, and then the absorbance was measured at 649 nm as a function of time within 120 h. La(NO_3_)_3_ (10 μM), Ce(NO_3_)_3_ (20 μM), Tb(NO_3_)_3_ (20 μM), and absolute ethanol were separately mixed with 4 mL chlorophyll mixture solution in equal volume of 1:1. Ultraviolet-visible spectra of these solutions and the absorbance were measured for 69 h using a spectrophotometer (Shimadzu UV-3900 UV-VIS Spectrophotometer, Tokyo, Japan). All the reactions concerning chlorophyll were conducted under dark conditions.

### Quantum Chemical Calculations

All the calculations were performed by applying the Gaussian 09 software (Gaussian 09) (Frisch et al., 2010) with density functional theory (DFT). Geometric optimizations were carried out by B3LYP functional with empirical dispersion. The 6-31G(d) basis set was adopted for C, N, O, H, and Mg, and the Stuttgart-Dresden (SDD) basis set was employed for La, Ce, and Tb. The solvent effect was taken into account by the polarizable continuum model (PCM) of the self-consistent reaction field (SCRF) procedure in ethanol. The vibrational frequencies were computed to confirm that the optimized structures had no imaginary frequency. The Gibbs free energies were calculated for the optimized structures at 298.15 K and 101 kPa. Furthermore, the electronic absorption spectra of optimized structures were simulated using the TDDFT with the Coulomb attenuated method (CAM)-B3LYP functional.

### Statistical Analysis

For statistical analysis, a one-way ANOVA was performed between treatment samples in three replications. To compare the seed germination rate, shoot length, root length, root activity, and total chlorophyll content of different treatments, data were analyzed using SPSS 26.0. The significant levels of difference for all the measured traits were calculated and means were compared by Duncan's multiple range test at a 5% level. Value of *P* smaller than or equal to 0.05 was considered statistically significant.

## Results

### Plant Seedling Growth

**Figure 1:**
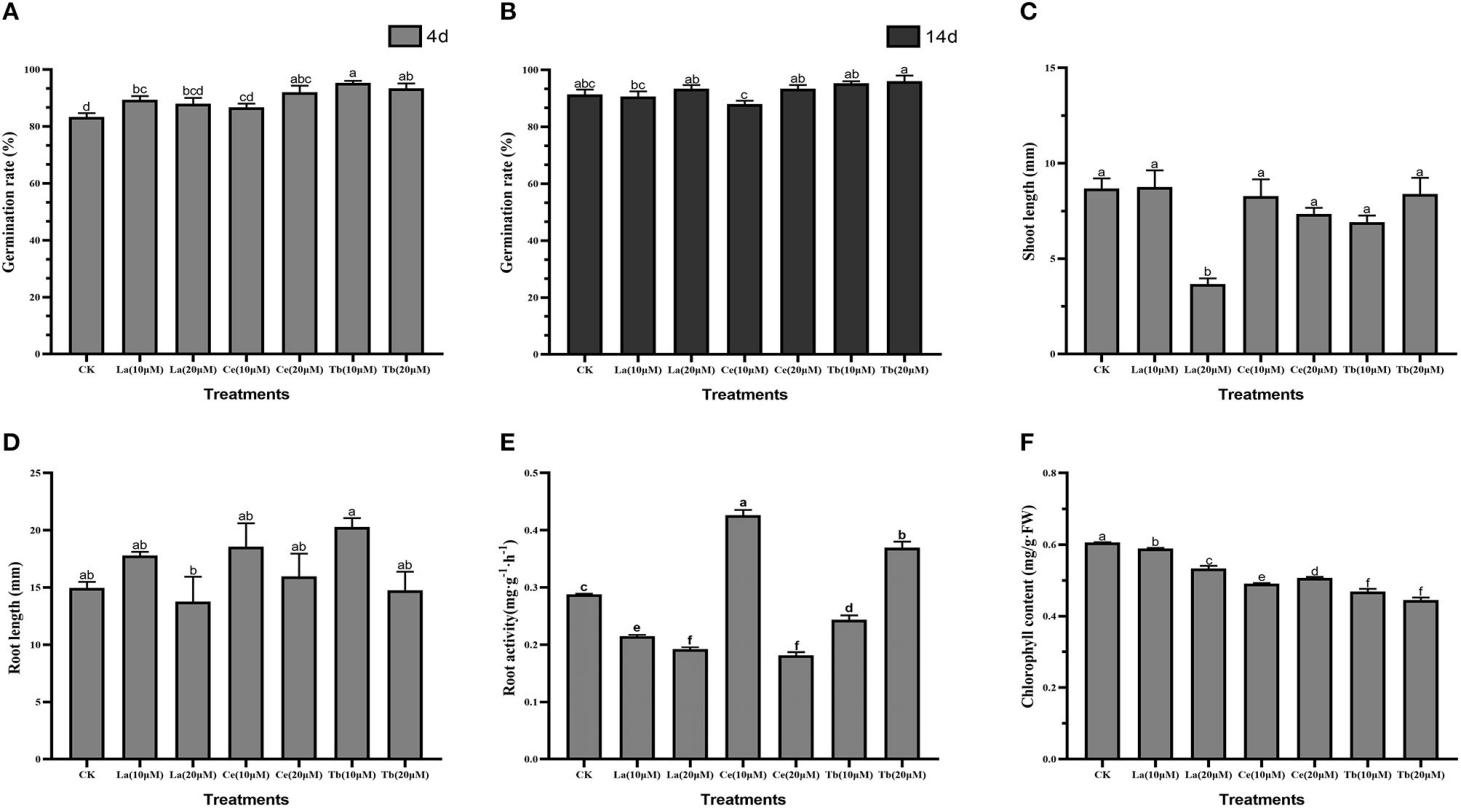
Effects of La, Ce, and Tb (10 and 20 μM) treatments on seed germination and seedling growth in Alfalfa. **(A)** Germination rate in 4 d, **(B)** germination rate in 14 d, **(C)** shoot length, **(D)** root length, **(E)** root activity, **(F)** chlorophyll content. Means with different letters are significantly different at *p* < 0.05.

To reveal the effects of treatments with La, Ce, and Tb on alfalfa seedling, seed germination rate, root length, shoot length, root activity, and chlorophyll content were measured. The results showed that treatments with the three REEs with 10 and 20 μM concentrations could speed up the germination of alfalfa, but had no significant effect on seed germination rate for the 14 days (Figures 1A,B).

As for shoot length, only 20 μM La treatment had a significant difference with the control, and it reduced the shoot length by 3.66 mm. For treatment with Ce, the increase in Ce concentration from 10 to 20 μM slightly shortened the shoot length, while treatment with Tb resulted in just the opposite (Figure 1C).

As for root length, the average root length of control was 14.98 mm. Compared with the control, the treatment with 20 μM La significantly reduced the root length, while the treatment with 10 μM Tb significantly elongated the root length. The average root lengths on treatments with 10 μM La, Ce, and Tb were 17.79, 18.56, and 20.29 mm, respectively, and the corresponding values were 13.77, 15.97, and 14.76 mm at the concentration of 20 μM. Compared with the control, all the treatments with 10 μM La, Ce, and Tb increased the root length, whereas all treatments with 20 μM La, Ce, and Tb decreased the root length, which showed the characteristic of “hormesis effect” (Figure 1D).

As for root activity, the value of the control was 0.286 mg/(g·h), and significant differences were found in all treatments. The two treatments with La significantly reduced the root activity of alfalfa. Compared to the control, the treatment with 10 μM Ce significantly increased the root activity by 43.7%, while the treatment with 20 μM Ce significantly decreased the root activity by 35.4%, which revealed the hormesis effect. On the contrary, the treatment with 10 μM Tb significantly decreased the root activity by 20.6%, whereas the treatment with 20 μM Tb significantly increased the root activity by 23.7% (Figure 1E).

Chlorophyll content was also an important index for above-ground growth. Compared with the control, all treatments with La, Ce, and Tb significantly decreased the chlorophyll content. The treatments with 10 μM La, Ce, and Tb decreased the chlorophyll content by 3.03, 18.34, and 24.33%, respectively, and the corresponding values were 9.54, 15.71, and 24.86% under 20 μM treatments. On the whole, the decrease in chlorophyll content was in the order of La < Ce < Tb (Figure 1F).

### *In vitro* Experiments

#### The Absorbance of Chlorophyll Mixture as a Function of Time

**Figure 2:**
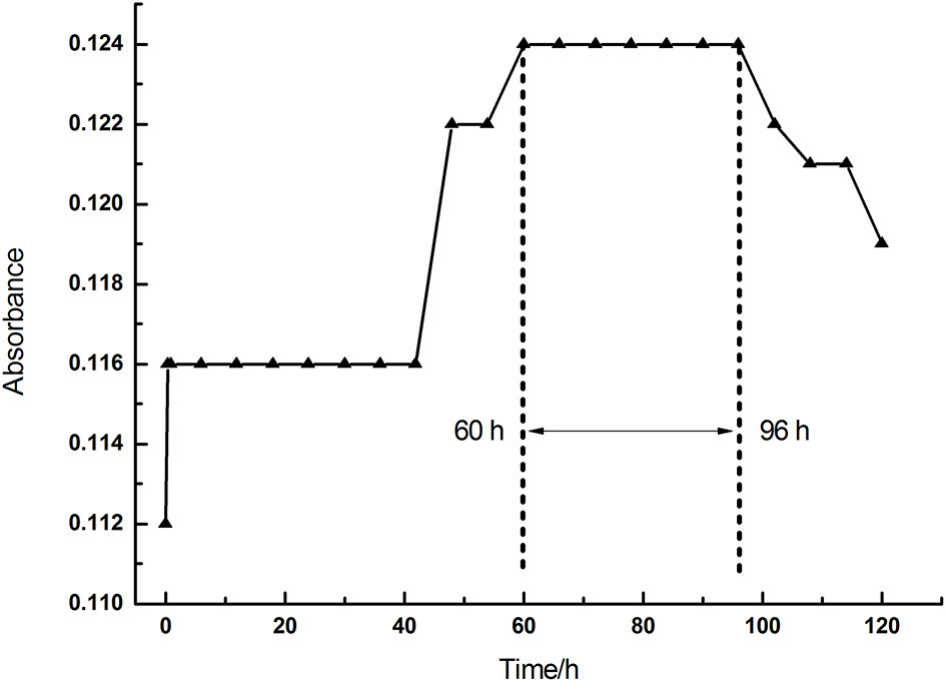
The absorbance of chlorophyll mixture as a function of time (0–120 h).

To sum up, we found that all treatments with La, Ce, and Tb reduced the chlorophyll content in alfalfa leaves *in vivo*. We conducted *in vitro* experiments to reveal how the treatments with La, Ce, and Tb affected the chlorophyll content. Firstly, the absorbance of the purchased chlorophyll mixture [Mg-chlorophyll a (MgCA) and Mg-chlorophyll b (MgCB)] in absolute ethanol was measured as a function of time. It can be seen in Figure 2 that the absorbance of chlorophyll mixture first increased from 0.112 to 0.124 within 60 h, and then reached a plateau between 60 and 96 h. Finally, it decreased after 96 h. Our *in vitro* experiments of REE treatments were performed at the plateau.

#### The Absorbance of Different Treatments *in vitro*

**Figure 3:**
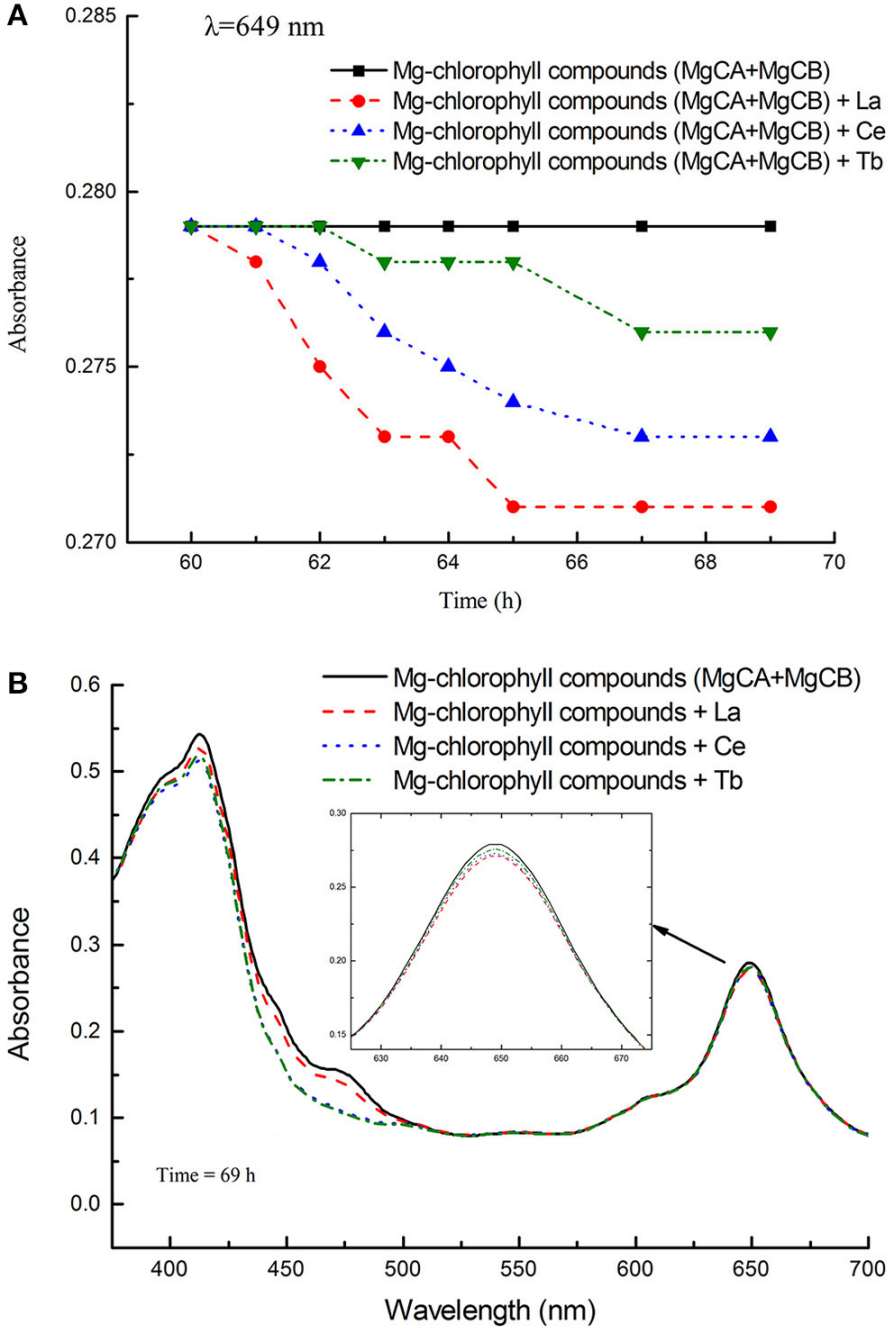
The absorbance of different treatments in the *in vitro* experiments. **(A)** The absorbance of the blank control (MgCA + MgCB), La(NO_3_)_3_ + MgCA + MgCB, Ce(NO_3_)_3_ + MgCA + MgCB, and Tb(NO_3_)_3_ + MgCA + MgCB treatments as a function of reaction time, **(B)** UV-vis spectra of the control (MgCA + MgCB), La(NO_3_)_3_ + MgCA + MgCB, Ce(NO_3_)_3_ + MgCA + MgCB, and Tb(NO_3_)_3_ + MgCA + MgCB treatments.

The absorbance and UV-vis spectra of chlorophyll mixture (MgCA + MgCB) and three treatments [La(NO_3_)_3_ + MgCA + MgCB, Ce(NO_3_)_3_ + MgCA + MgCB, and Tb(NO_3_)_3_ + MgCA + MgCB] were measured. The absorbance was measured at 649 nm as a function of time. In Figure 3A, it can be seen that all treatments with La, Ce, and Tb decreased the absorbance at 649 nm, and the decrease was in the order of La > Ce > Tb. In Figure 3B, the UV-vis spectra of chlorophyll mixture and treatments with La, Ce, and Tb were very similar, and the UV-vis spectra had two strong absorption peaks in the visible region, which appeared at about 413 and 649 nm, respectively. Moreover, at both absorption peaks, the absorbance of the chlorophyll mixture was always the largest.

#### Quantum Chemical Calculations

All the treatments with La, Ce, and Tb decreased the absorbance of the chlorophyll mixture *in vitro*, which was consistent with *in vivo* experiments. This situation indicated that La, Ce, and Tb might react with chlorophyll directly, and form lanthanides-chlorophyll. Under this circumstance, we further investigated the possible reactions between chlorophyll and La, Ce, and Tb with quantum chemical calculations.

**Figure 4:**
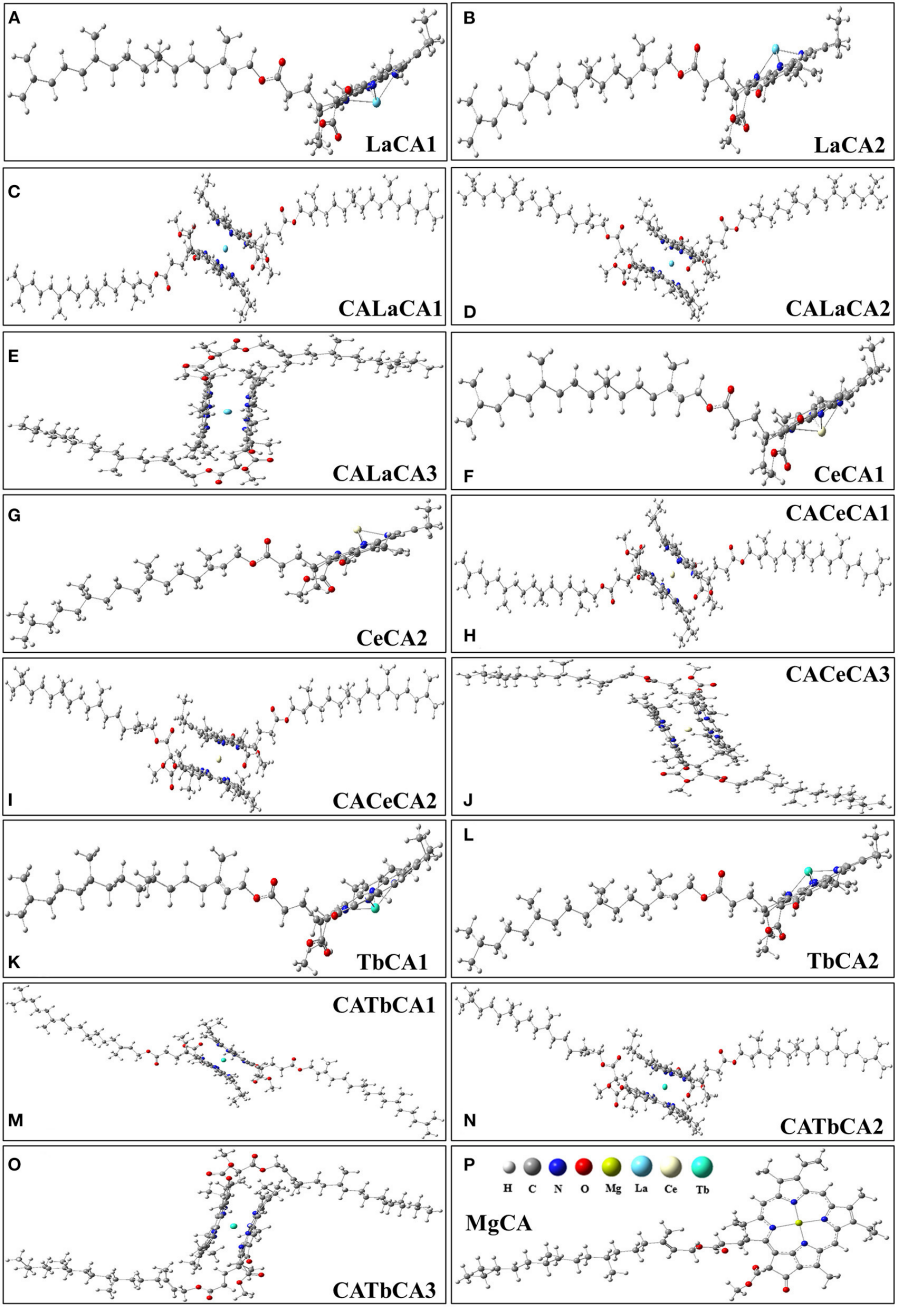
Optimized structures of products for the reactions between La, Ce, Tb, and chlorophyll a. **(A,B)** lanthanum mono-chlorophyll a, **(C–E)** lanthanum bis-chlorophyll a, **(F,G)** cerium mono-chlorophyll a, **(H–J)** cerium bis-chlorophyll a, **(K,L)** terbium mono-chlorophyll a, **(M–O)** terbium bis-chlorophyll a, **(P)** chlorophyll a.

In quantum chemical calculations, for the sake of simplicity, we only considered chlorophyll a. In the *in vitro* experiments, La, Ce, and Tb might replace Mg in chlorophyll a, and form lanthanides-chlorophyll. We systematically studied the possible products of reactions between chlorophyll a (MgCA) and La, Ce, and Tb. We found that there were two kinds (lanthanides mono-chlorophyll a and lanthanides bis-chlorophyll a) and five possible compounds that were formed by REE and chlorophyll a, and we named them RECA1, RECA2, CARECA1, CARECA2, and CARECA3 (RE = La, Ce, and Tb). In RECA1, REE is coordinated with one porphyrin ring, and REE and the hydrocarbon tail are on different sides of the porphyrin ring. In RECA2, REE is coordinated with one porphyrin ring, and REE and the hydrocarbon tail are on the same side of the porphyrin ring. In CARECA1, REE is sandwiched by two porphyrin rings, and two long hydrocarbon tails are both on the outside. In CARECA2, REE is sandwiched by two porphyrin rings, and one long hydrocarbon tail is on the outside and the other is on the inside. In CARECA3, REE is sandwiched by two porphyrin rings, and both the long hydrocarbon tails are on the inside. The optimized structure is shown in Figure 4.

**Figure 5:**
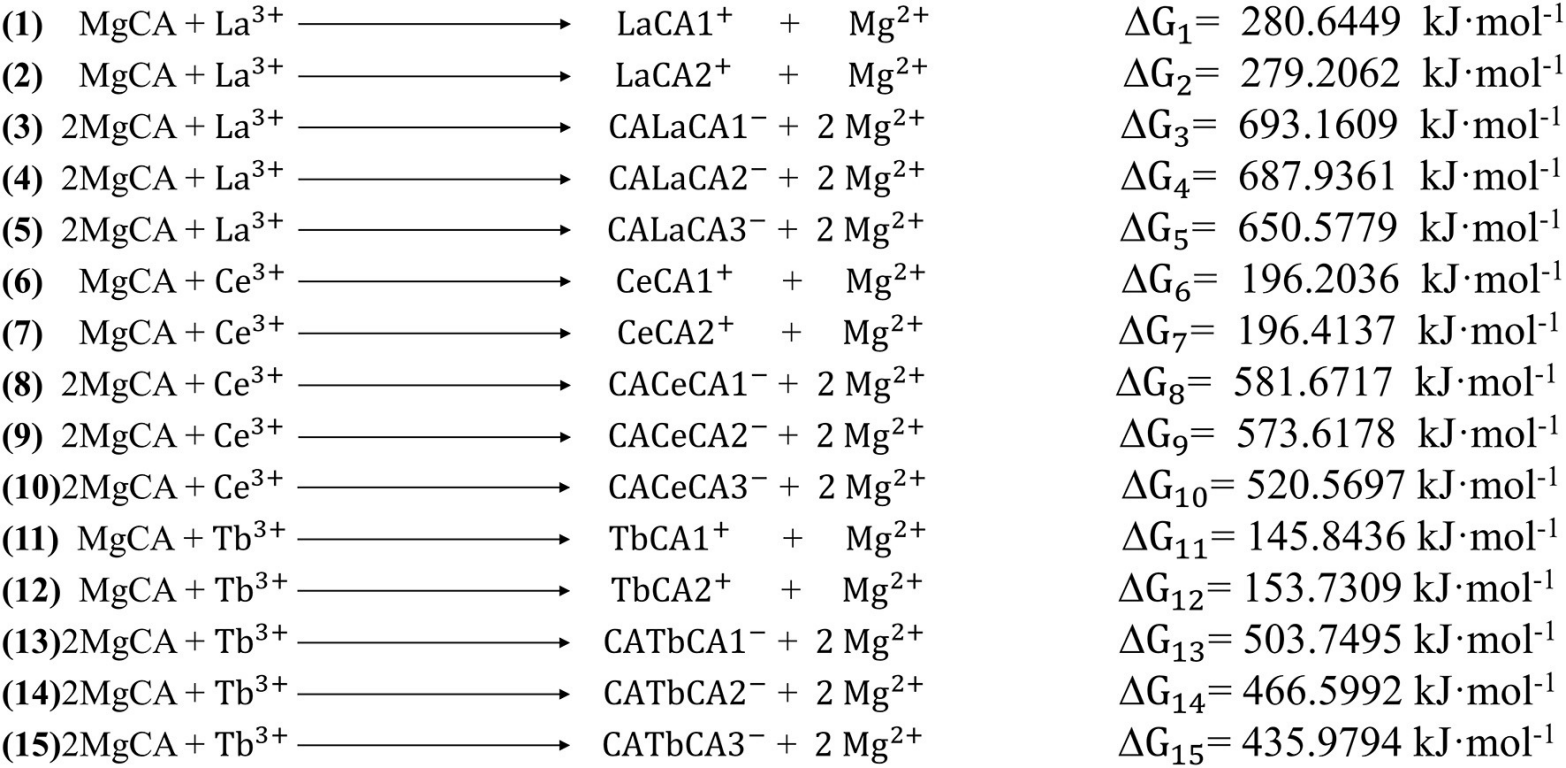
The changes in Gibbs free energy for the 15 possible reactions.

For these optimized structures, it was necessary to evaluate which compound was more stable and whether the given chemical reaction was thermodynamically possible. Therefore, the Gibbs free energies of these optimized structures at 298.15 K and 101 kPa in ethanol were calculated, and these are listed in Table 1. Furthermore, the changes in Gibbs free energy for the chemical reactions between different compounds were also calculated. There are 15 chemical reactions between chlorophyll a and REEs (La, Ce, and Tb), and the changes in the Gibbs free energy for these reactions are illustrated in Figure 5.

**Table 1:** Gibbs free energies of optimized structures.

**Compounds**		**Gibbs free energy (kJ·mol^**−1**^)**
1	Mg^2+^	−524714.6869
2	La^3+^	−1143186.1205
3	Ce^3+^	−1246719.1896
4	Tb^3+^	−95035.9140
5	MgCA	−7702602.4755
6	LaCA1	−8320793.2642
7	LaCA2	−8320794.7029
8	CALaCA1	−15498268.5368
9	CALaCA2	−15498273.7615
10	CALaCA3	−15498311.1198
11	CeCA1	−8424410.7746
12	CeCA2	−8424410.5645
13	CACeCA1	−15601913.0951
14	CACeCA2	−15601921.1490
15	CACeCA3	−15601974.2000
16	TbCA1	−7272777.8590
17	TbCA2	−7272769.9717
18	CATbCA1	−14450307.7417
19	CATbCA2	−14450344.8920
20	CATbCA3	−14450375.2118

Gibbs free energy is a thermodynamic function introduced to determine the direction of the chemical reaction. The smaller the change in Gibbs free energy, the greater the possibility for the reaction to occur. From Figure 5, it was seen that all the changes in Gibbs free energy were positive, and their values were in the range of 145–693 kJ·mol^−1^. Generally speaking, for the same REE, the Gibbs free energy changes for MgCA + REE reactions were much smaller than those for 2MgCA + REE reactions. As a result, lanthanides mono-chlorophyll a compounds were much easier to be formed than lanthanides bis-chlorophyll a compounds. For different REEs, the Gibbs free energy change for the same substitution reaction was different, and its value decreased in the order of La > Ce > Tb, which indicated that it was easiest for Tb to form lanthanides-chlorophyll a compounds. Moreover, for different REEs, the most stable lanthanides-chlorophyll a compound was also different. The most stable compounds for La, Ce, and Tb were LaCA2, CeCA1, and TbCA1, respectively.

#### Electronic Absorption Spectrum

Electronic absorption spectra of possible lanthanides-chlorophyll a compounds were simulated to reveal how the chlorophyll structure affected the absorbance.

**Figure 6:**
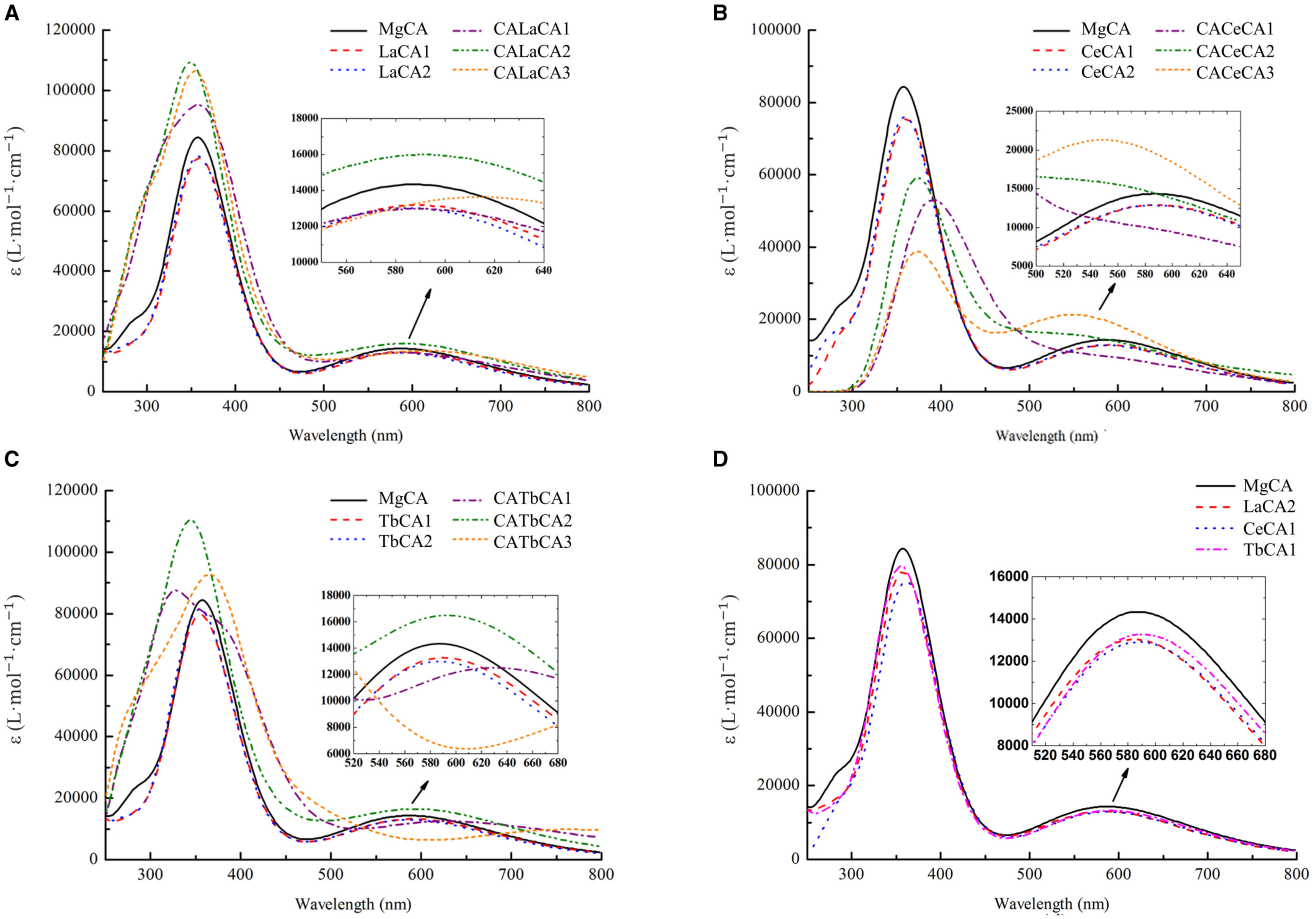
Simulated electronic absorption spectra of different compounds (La, Ce, and Tb) in ethanol. **(A)** Mg-chlorophyll a and La-chlorophyll a, **(B)** Mg-chlorophyll a and Ce-chlorophyll a, **(C)** Mg-chlorophyll a and Tb-chlorophyll a, **(D)** Mg-chlorophyll a and lanthanides mono-chlorophyll a (LaCA2, CeCA1 and TbCA1).

As shown in Figures 6A–C, the electronic absorption spectrum of MgCA had two absorption peaks at 358 and 584 nm, respectively. Compared to our UV-vis spectrum, these two peaks were blue-shifted by 53 and 65 nm, respectively. Indeed, our electronic absorption spectrum of MgCA was consistent with the previous calculations (Chen et al., 2003; He et al., 2020).

The electronic absorption spectra of lanthanides-chlorophyll a compounds still had two absorption peaks, which were similar to the MgCA peaks. However, the absorption intensity changed a lot. For lanthanides mono-chlorophyll a compounds, the two peaks at the short- and long-wavelength bands, respectively, were lower than those of MgCA, which demonstrated that the formation of lanthanides mono-chlorophyll a compounds reduced the absorbance of chlorophyll. For lanthanides bis-chlorophyll a compounds, at the short-wavelength band, both La and Tb enhanced the absorption intensity, whereas Ce weakened the absorption intensity, and at the long-wavelength band, all the compounds lowered the absorption intensity, except CALaCA2, CACeCA3, and CATbCA2.

The electronic absorption spectra of the most stable compounds for La, Ce, and Tb are depicted in Figure 6D. It was seen that the absorption intensity of MgCA was the largest, and the absorption intensity of TbCA1 was larger than that of LaCA2 and CeCA1.

## Discussion

In this study, we observed that treatments with REEs quickened the germination of alfalfa seeds, and the treatments with REEs at low concentration promoted the root growth of the seedling. The root is the main organ that absorbs water and nutrients. For the alfalfa seeds, the REEs treatments at appropriate concentration could enhance root activity. This phenomenon is consistent with the effect of REEs on root length reported by others (Diatloff et al., 1995). However, whether REEs are beneficial depends on the crop genotypes and the time of measurement (García-Jiménez et al., 2017).

In our experiments, La(NO_3_)_3_ (10 and 20 μM), Ce(NO_3_)_3_ (10 and 20 μM), and Tb(NO_3_)_3_ (10 and 20 μM) were used to treat the alfalfa seeds, and distilled water was used as the control. So, the changes in physiological parameters were caused by both REEs and nitrate ions in our treatments with REEs. However, the response concentrations of plants to REEs and nitrate ions are different. Nitrate plays a role in nutrition and signaling in many plants, and it needs a relatively high concentration to stimulate or promote plant growth. For example, KNO_3_ concentrations from 1 to 25 mM did not significantly affect the germination rate and speed of *Senecio coincyi* (Schnadelbach et al., 2016). Duermeyer et al. (2018) reported that the response concentration of various plants to nitrate was usually above 0.1 mM, which is much higher than that adopted in our experiments. In plants, the high-affinity nitrate transport system is used to absorb low concentrations of exogenous nitrogen. In alfalfa, the response concentration of exogenous nitrogen reported in the latest research was 5 mM (Zou et al., 2019), and according to another study, the response concentration of exogenous nitrogen was 0.2 mM in the soil (Wang et al., 2020). The response concentration of nitrate as exogenous nitrogen in alfalfa was reported at the mM level. According to the International Seed Testing Association (2017), even after 14 days of growing the seeds on paper, they did not lack nitrogen. Moreover, in our experiments, the seeds imbibed only 5 mL of REEs solution on the first day and were replenished with a specific quantity of water every day. The amount of nitrogen from an external source was extremely little. REEs usually have a positive impact on seed germination and growth at relatively low concentrations. For instance, Agathokleous et al. (2018) summarized the hormesis dose-response induced by lanthanum in plants and found that the “hormesis effect” commonly occurred at a concentration of few tens of μM. So, the nitrate ion in our experiments may have a weak impact on alfalfa germination and growth. As a result, the REEs play a major role in alfalfa germination and growth based on our experiments.

The leaf chlorophyll content is an important index to reflect leaf physiological status. Wei et al. (2005) described that REEs are bound to chlorophyll and found that the REEs may replace magnesium in chlorophyll. Hong et al. (2002) studied the effect of Ce on chlorophyll of spinach and found that Ce could enter chloroplast and combine with chlorophyll. Our *in vivo* experiments also observed significant changes in the chlorophyll content in alfalfa leaves under different concentrations of REEs. However, there are two reasons for the change in chlorophyll content: (1) the change in total chlorophyll content; and (2) the change in chlorophyll structure. Therefore, we further carried out *in vitro* experiments and quantum chemical calculations.

The chlorophyll content was significantly decreased by REE treatments *in vivo*. This situation demonstrated that REEs might suppress chlorophyll production or change the chlorophyll form in alfalfa. This conclusion was evidenced in our *in vitro* experiments, which showed that La, Ce, and Tb treatments reduced the absorbance of chlorophyll by reacting with chlorophyll. Our quantum chemical calculations further predicted that La, Ce, and Tb could replace Mg in chlorophyll a, and form lanthanides mono-chlorophyll a and lanthanides bis-chlorophyll a compounds. The changes in Gibbs free energy for these substitution reactions revealed that lanthanides mono-chlorophyll a compounds were much easier to be formed than lanthanides bis-chlorophyll a compounds. The simulated electronic absorption spectra of lanthanides mono-chlorophyll a compounds showed that the replacements of Mg by La, Ce, and Tb lowered the absorption intensity of chlorophyll. So, we speculated that the formation of lanthanides-chlorophyll decreased the chlorophyll content in alfalfa. Our conclusion was consistent with the results of Hong et al. (2002), who found that lanthanum could replace magnesium ions to form spinach chlorophyll.

The chlorophyll content decreased in the order of La < Ce < Tb *in vivo*. However, the absorbance decreased in the order of La > Ce > Tb *in vitro*. This contradiction was caused by the degree of difficulty in the substitution reactions. According to the change in Gibbs free energy, the possibility of replacing Mg by REEs followed in the order of La < Ce < Tb. It was clear that although replacing Mg by La showed the maximum reduction in absorbance, Tb could replace the largest amount of Mg. Therefore, more Mg could be replaced by Tb in the 14 days, and Tb treatment had the lowest absorbance and chlorophyll content *in vivo*.

It was reported that REEs may affect plant cells. Exogenous rare earth elements (La, Ce, and Tb) were immobilized on the plasma membrane by AGPs in the form of nanoparticles, and then irreversibly changed the molecules with the increase in concentration, thus damaging the cells (Wang et al., 2019). However, a low concentration of REEs only affected the normal photosynthesis of plants (Wang, L., et al., 2014). For example, most of the REEs with low concentration adhered to the cell wall (Ye et al., 2008) or bound to the cell membrane (Ouyang et al., 2003), and the free radical reaction was in equilibrium (Wang et al., 2009), which increased the stability of the membrane (Gill et al., 1981) and activity of the corresponding enzyme (Hu et al., 2016a), thus stimulating and compensating the chlorophyll content. On the other hand, a high concentration of REEs entered the cell and adhered to the chloroplast, changing the ultrastructure of the chloroplast (Hu et al., 2016b). Although LREEs and HREEs enter the cells in a similar way (Wang L. H. et al., 2014), they have different beneficial effects on different genotypes of plants under different culture conditions. García-Jiménez et al. (2017) found that on comparing with the control, chlorophyll content of pepper decreased after 15 days of hydroponics, but increased after 30 days of hydroponics. Kotelnikova et al. (2020) reported that there was no significant difference in decline in chlorophyll content, except for La (100 mg/kg) group, when La, Ce, and Nd were applied to the soil. It means that different plants have different concentration dependence of REEs.

## Conclusions

The results showed that treatments with REEs (La, Ce, and Tb) had an effect on the growth of alfalfa seedlings. Treatments with REEs quickened the germination of alfalfa seeds, and the treatments at appropriate concentrations promoted the growth of roots and increased root activity. Moreover, the chlorophyll content was significantly reduced under treatments with REEs, and its decrease was in the order of La > Ce > Tb. Quantum chemical calculations predicted that La, Ce, and Tb could replace Mg in chlorophyll a, and form lanthanides mono-chlorophyll a and lanthanides bis-chlorophyll a compounds. The possibility of replacing Mg by REEs followed in the order of La < Ce < Tb. Therefore, in alfalfa, more Mg could be replaced by Tb in the 14 days, and the treatments with Tb had the lowest absorbance and chlorophyll content. Furthermore, REE fertilizers could result in the enrichment of rare earth elements in leaves.

## Data Availability Statement

The raw data supporting the conclusions of this article will be made available by the authors, without undue reservation.

## Author Contributions

XH: conceptualization, funding acquisition, supervision, and validation. KS, JG, SL, YS, HS, and BA: data curation and investigation. XH and KS: formal analysis, software, writing—original draft, and writing—review and editing. XH, KS, JG, SL, YS, HS, and BA: methodology. TH and XH: project administration and resources. All authors contributed to the article and approved the submitted version.

## Funding

This work was supported by the Tibet Finance Department Project (Grant No. XZ202001ZY0016N), the Key Industry Innovation Chain in Shaanxi Province of China (Grant No. 2019ZDLNY05-02), the National Natural Science Foundation of China (Grant No. 31502005), and the National College Students' Science and Technology Innovation Project (Grant No. S202010712001). The funders had no role in study design, data collection, and analysis.

## Conflict of Interest

The authors declare that the research was conducted in the absence of any commercial or financial relationships that could be construed as a potential conflict of interest.

## Publisher's Note

All claims expressed in this article are solely those of the authors and do not necessarily represent those of their affiliated organizations, or those of the publisher, the editors and the reviewers. Any product that may be evaluated in this article, or claim that may be made by its manufacturer, is not guaranteed or endorsed by the publisher.

## Abbreviations

La: lanthanum
Ce: cerium
Tb: Terbium
REEs: rare earth elements
LREE: light rare earth elements
HREE: heavy rare earth elements
CME: clathrin-mediated endocytosis
AGPs: arabinogalactan proteins
FW: fresh weight
TTC: triphenyl tetrazolium chloride
MgCA: Mg-chlorophyll a
MgCB: Mg-chlorophyll b
DFT: density functional theory
SDD: Stuttgart-Dresden
PCM: polarizable continuum model
SCRF: self-consistent reaction field
TDDFT: time-dependent density functional theory.
